# Sociolinguistic Markers of Friendship Among Persons With Dementia in Long-Term Care

**DOI:** 10.1093/geroni/igaa057.1911

**Published:** 2020-12-16

**Authors:** Pamela Saunders

**Affiliations:** Georgetown University, Washington, District of Columbia, United States

## Abstract

Sociolinguistics and discourse analysis provide tools through which to examine how friendship is socially constructed through language and communication. Research on social isolation and loneliness reveals the importance of social interaction on the psychological and physical health of older adults. Given that linguistic, communicative, and functional abilities decline as dementia progresses, it is challenging to identify markers of friendship. The Friendship Project is an ethnographic study of social interaction among persons with dementia living in a long-term care setting. The data are from transcripts and field-notes of social interactions among residents with a range of cognitive impairments over a six-month time period. Results reveal that persons with dementia employ specific linguistic features such as narrative, evaluation, evidentials, and pronominal reference to make meaning and create relationships over time. Practical implications will be discussed.

